# Short- and long-term outcomes of percutaneous coronary intervention in patients with low, intermediate and high ejection fraction

**Published:** 2008-02

**Authors:** M Alidoosti, M Salarifar, AMH Zeinali, SE Kassaian, MR Dehkordi, MS Fatollahi

**Affiliations:** Tehran Heart Centre, Medical Sciences, University of Tehran, Tehran, Iran; Tehran Heart Centre, Medical Sciences, University of Tehran, Tehran, Iran; Tehran Heart Centre, Medical Sciences, University of Tehran, Tehran, Iran; Tehran Heart Centre, Medical Sciences, University of Tehran, Tehran, Iran; Tehran Heart Centre, Medical Sciences, University of Tehran, Tehran, Iran; Tehran Heart Centre, Medical Sciences, University of Tehran, Tehran, Iran

## Abstract

**Background:**

Reduced ejection fraction (EF) has previously been shown to be a risk factor for adverse outcomes in patients undergoing percutaneous coronary intervention (PCI). However, with the advent of stents, procedural complications and restenosis rates have reduced dramatically. The aim of this study was to assess the association between left ventricular (LV ) ejection fraction and in-hospital and longterm outcomes using a prospective registry.

**Methods:**

After exclusion of patients with acute myocardial infarction (MI) and those with missing data on left ventricular ejection fraction, 2 030 patients undergoing PCI between March 2002 and 2004 remained in our prospective registry. Patients were divided into three categories: group 1: EF ≤ 40% (*n* = 293), group 2: EF = 41−49% (*n* = 268) and group 3: EF ≥ 50% (*n* = 1 469). The frequency of in-hospital and follow-up outcomes between groups was compared using appropriate statistical methods.

**Results:**

Stents were used for over 85% of the patients in each group. The mean EF ± SD in the lowest to highest EF groups was 35.8 ± 5.4%, 45.5 ± 1.6% and 57 ± 5.7%, respectively. The angiographic and procedural success rates were 91.8, 92.1 and 94.1%, (*p* = 0.16); and 91.1, 90.3 and 92.9%, (*p* = 0.09), respectively. The respective cumulative major adverse cardiac events (MACE) and cardiac death rates at follow-up were 5.8, 2.2 and 3.3% (*p* = 0.04) and 2, 0.4 and 0.3% (*p* = 0.02), respectively. The hazards ratio (95% CI) for MACE and cardiac death in the lowest versus highest EF groups were 2.07 (1.03−4.16) and 5.49 (1.29−23.3).

**Conclusions:**

Patients with significant left ventricular dysfunction had higher long-term major adverse cardiac events and cardiac death rates. Even the use of newer techniques such as stenting did not compensate for this.

## Summary

Although mortality from coronary artery disease is declining, the incidence of associated heart failure is rising.^1^ The prognostic significance of left ventricular (LV) dysfunction in patients with coronary artery disease has been demonstrated in many studies.^2^-^6^ Similarly, reduced ejection fraction (EF) has previously been shown to be a risk factor for adverse in-hospital and long-term outcomes in patients undergoing percutaneous coronary intervention (PCI).^7^-^13^

It is unclear whether coronary artery bypass grafting (CABG) provides a more appropriate choice for revascularisation than does PCI in patients with LV dysfunction. Previous studies have shown that patients with left ventricular ejection fraction (LVEF) ≤ 25% showed improved results with surgery.^14^ CABG may also be the best treatment for patients with LV dysfunction and diabetes. However, no survival benefit has been noted for CABG over percutaneous transluminal coronary angioplasty (PTCA) in non-diabetic patients.^15^

Recent technical advances in the field of interventional cardiology and an increase in operator experience have resulted in substantial increases in the complexity and volume of the procedures, and improvement of acute and long-term success rates. With the advent of stents, outcomes in terms of periprocedural complications and restenosis have been much better than previously reported for PTCA alone.^16^, ^17^

In the present study, we aimed to assess the association between LVEF and in-hospital and long-term outcomes in the current catheter-based practice, using a prospective registry of 2 591 patients who underwent PCI between March 2002 and 2004. Also, as a subgroup study, we analysed these outcomes in a group of patients who had been treated with only stenting techniques.

## Methods

All patients treated with PCI at our centre between March 2002 and 2004 were entered into a prospective registry. The first wave of this registry consisted of 2 591 patients. We excluded 73 patients in whom the primary reason for revascularisation was acute myocardial infarction within the preceding 48 hours. Quantitative data on LV function were available for 2 030 (80.6%) of the patients, using contrast ventriculography, LV angiography and echocardiography. These patients were divided into three groups according to LVEF: group 1: EF ≤ 40%; group 2: EF 5 41−49%; and group 3: EF ≥ 50%.

Baseline demographic and clinical details, angiographic and procedural characteristics, and in-hospital outcomes were obtained by research physicians. Follow-up outcomes were obtained by cardiologists in clinics at one and six months, and once a year thereafter, or by formal telephone interviews in patients who had given consent to participate in follow-up programmes after discharge from hospital. Computer operators entered all the data into a computerised database. Consent to collect post-discharge follow-up data was not obtained for 214 (10.5%) of the patients who survived hospitalisation. Of these, 33 (11.3%) were in EF group 1, 33 (12.3 %) were in EF group 2, and 148 (10.1%) were in EF group 3.

Patients received 325 mg of aspirin before the procedure, and it was continued indefinitely. Coronary stenting was performed by standard methods. After stent placement, ticlopidine (250 mg twice daily) or clopidogrel (75 mg once daily) was given routinely for four weeks to patients with bare metal stents, and six to 12 months with drug-eluting stents. Angiographic findings such as vessel dimensions, pre- and post-procedural stenoses, lesion length and thrombolysis in myocardial infarction (TIMI) flow grade were determined by visual estimation using the guiding catheter as a reference object for calibration. The angiographic characteristics were also further analysed by an independent interventional cardiologist not involved in the procedure.

## Definitions

Angina symptoms were defined according to the classification of the Canadian Cardiovascular Society.^18^ Lesion types were noted according to the American College of Cardiology/American Heart Association (ACC/AHA) lesion characteristics classification.^19^ Q-wave MI was defined as the presence of new Q waves in a post-procedure electrocardiogram, with a threefold increase in MB fraction of creatinine kinase. Non-Q-wave MI was defined as a three-fold increase in MB fraction of creatinine kinase without the development of new Q waves.^20^

Angiographic success was defined as residual stenosis below 20% for stenting and below 50% for balloon angioplasty plus normal TIMI 3 flow. Procedural success was defined as angiographic success without major complications (death, MI, emergency bypass surgery or PCI) during hospitalisation. Major adverse cardiac events (MACE) were defined as the presence of cardiac death, non-fatal MI, or target vessel revascularisation (TVR) during the follow-up period. TVR was defined as ischaemia-driven repeat percutaneous intervention or bypass surgery of the target vessel. Target lesion revascularisation (TLR) was defined as ischaemia-driven repeat percutaneous intervention of the target lesion or bypass surgery of the target vessel.^21^

This study was approved by the Tehran Heart Centre Ethics Committee. Informed consent was obtained from all patients before enrolment into this study.

## Statistics

The trends for differences in baseline characteristics and inhospital outcomes across the groups were compared by the Mantel-Haenszel and Kendall’s tau rank correlation tests. Multivariate Cox proportional hazards models were developed for comparison of the rates of MACE and its constructing components (cardiac death, non-fatal MI and TVR) in followup, identification of variables associated with each outcome, and calculation of adjusted risk ratios. Event-free survival curves were drawn using the Kaplan-Meier method. The log rank was used to test for differences between survivals.

A multivariate logistic regression model was constructed to compare the in-hospital outcomes, to determine the independent predictors, and to calculate the adjusted risk ratios. The risk ratios and 95% confidence intervals for events were calculated for each of the first EF groups (EF ≤ 40% and EF 5 41−49%) compared to the third group (EF ≥ 50%). A 0.15 level of significance for baseline and procedural characteristics in univariate analysis was considered suitable as a cut-off point for entering into multivariate models. We also analysed EF as a continuous variable. Univariate analyses were performed with SPSS software version 13. Multivariate analyses were conducted with SAS software version 9.1.

## Results

Among the 293 patients (14.4%) with an EF ≤ 40% (group 1), the mean EF was 35.8 ± 5.4% (range, 15−40%). Two hundred and sixty-eight patents (13.2%) had an EF between 41 and 49%, with a mean of 45.5 ± 1.6%. In the remaining 1 469 patients (72.4%) with EF ≥ 50% (group 3), mean EF was 57 ± 5.7% (range, 50−80%). The three groups had significant differences in baseline characteristics (Table 1). The lower the EF value, the more likely patients were to be men (*p* < 0.001) and to have a history of MI (*p* < 0.001). Patients in the highest EF value were less likely to be smokers (*p* = 0.03) and more likely to have a history of hypertension (*p* < 0.001).

**Table 1.** Baseline Characteristics In Patients With EF ≤ 40%, EF 5 41−49% And EF ≥ 50%

	*Group 1 (EF ≤ 40%; n = 293)*	*Group 2 (EF = 41−49%; n = 268)*	*Group 3 (EF ≥ 50%; n = 1469)*	
Variable	n (%)	n (%)	n (%)	p-value
Age (mean ± SD)	56.14	55.15	56.08	0.52
Male	233 (79.5)	195 (72.8)	1014 (69.0)	**< 0.001**
Positive family history	65 (22.2)	61 (22.8)	311 (21.2)	0.60
Smoking	125 (42.7)	117 (43.7)	548 (37.3)	**0.03**
Hyperlipidaemia	128 (43.8)	102 (38.5)	691 (47.4)	**0.07**
Hypertension	84 (28.8)	82 (30.9)	554 (38.0)	**< 0.001**
Diabetes mellitus	69 (23.6)	58 (21.9)	315 (21.6)	0.47
Unstable angina	77 (26.3)	95 (35.4)	499 (34.1)	0.3
Previous MI	198 (67.6)	138 (51.5)	419 (28.5)	**< 0.001**
Previous CVA	3 (1.0)	1 (0.4)	10 (0.7)	0.69
Previous PCI	18 (6.1)	18 (6.7)	74 (5.0)	0.3
Previous CABG	13 (4.4)	5 (1.9)	37 (2.5)	**0.14**

MI: myocardial infarction, CVA: cerebrovascular accident, PCI: percutaneous coronary intervention, CABG: coronary artery bypass grafting. The *p*-values of variables entered into multivariate model are printed in bold.

Patients with the lowest EF value were also more likely to have type B2/C lesions in one or more coronary arteries (67.9 vs 63.6 and 56.5%, *p* < 0.001). Total occlusions in one or more coronary arteries were more frequent in the two lowest EF groups compared to the highest one (18.1 and 19% vs 11.5%, *p* < 0.001). Moreover, the lower the EF value, the more likely lesions were to be longer. Stents were used for over 85% of patients in each group and the most frequently used technique was stenting after predilation. Drug-eluting stents were less frequently used for patients with the lowest EF (*p* = 0.03). However, there was no significant difference across the groups in lengths and diameters of stents (Tables 2, 3).

**Table 2.** Angiographic And Lesion Characteristics In Patients With EF ≤ 40%, EF 5 41−49% And EF ≥ 50%

	*Group 1 (EF ≤ 40%; n = 293)*	*Group 2 (EF = 41−49%; n = 268)*	*Group 3 (EF ≥ 50%; n = 1469)*	
Variable	n (%)	n (%)	n (%)	p-value
Type B2/C	**182 (67.9)**	**159 (63.6)**	779 (56.5)	**0.001**
Proximal	114 (38.9)	100 (37.3)	576 (39.2)	0.79
Diffuse	84 (28.7)	76 (28.4)	360 (24.5)	0.08
Bifurcation	27 (9.2)	25 (9.3)	105 (7.1)	0.14
Angulated (< 45°)	24 (8.2)	16 (6.0)	115 (7.8)	0.89
Thrombus	11 (3.8)	9 (3.4)	38 (2.6)	0.23
LAD territory	200 (68.3)	177 (66.0)	918 (62.5)	**0.04**
Total	53 (18.1)	51 (19.0)	169 (11.5)	**< 0.001**
Multivessel disease	151 (57.4)	113 (57.1)	707 (54.1)	0.26
Lesion length (mm)*	18.42 ± 8.82	17.68 ± 7.98	16.71 ± 7.78	**0.001**
RVD (mm)*	2.98 ± 0.42	2.98 ± 0.43	2.99 ± 0.37	0.76

LAD: left anterior descending artery, *mean ± SD. The *p*-values of variables entered into multivariate model are printed in bold.

**Table 3.** Procedural Data And In-Hospital Outcomes In Patients With EF ≤ 40%, EF 5 41−49% And EF ≥ 50%

	*Group 1 (EF ≤ 40%; n = 293)*	*Group 2 (EF = 41−49%; n = 268)*	*Group 3 (EF ≥ 50%; n = 1469)*	
Variable	n (%)	n (%)	n (%)	p-value
Stent use	245 (85.7)	223 (86.8)	1270 (88.3)	0.42
Drug-eluting stents	19 (8.4)	34 (16)	179 (15)	**0.03**
Stent length (mm)*	18.25 ± 5.75	18.71 ± 6.34	18.41 ± 6.39	0.21
Stent diameter (mm)*	3.03 ± 0.37	2.99 ± 0.33	3.01 ± 0.34	0.92
In-hospital outcomes				
Angiographic success	269 (91.8)	246 (92.1)	1381 (94.1)	0.16
Procedural success	267 (91.1)	241 (90.3)	1363 (92.9)	0.09
Cardiac death	1 (0.3)	3 (1.1)	1 (0.1)	0.08
Q-wave MI	0	1 (0.4)	2 (0.1)	0.84
Non-Q-wave MI	1 (0.4)	2 (0.8)	17 (1.2)	0.18

MI: myocardial infarction, *mean ± SD. The *p*-values of variables entered into multivariate model are printed in bold.

## In-hospital outcomes

The Mantel Haenszel test showed no statistically significant trend across the three groups for the angiographic and procedural success rates (*p* = 0.16; *p* = 0.09, respectively) (Table 3). In multivariate logistic regression models, bifurcation and total occlusions were associated with lower acute procedural success rates (OR 5 0.18, 95% CI 5 0.11−0.31, *p* < 0.001, and OR 5 0.4, 95% CI 5 0.23−0.68, *p* < 0.001, respectively). The same factors plus diffuse lesions were independent predictors of procedural success rate (OR = 0.22, 95% CI = 0.14−0.37, *p* < 0.001; OR = 0.44, 95% CI = 0.27−0.73, *p* = 0.001, and OR = 0.62, 95% CI = 0.4−0.97, *p* = 0.03, respectively).

Cardiac death rate was higher in patients who had EF between 41 and 49% (1.1%). The corresponding values were 0.3% in group 1 and 0.1% in group 3 (*p* = 0.006). No patient underwent emergent CABG in the hospital. The rates of Q- and non-Q-wave MI were also similar for all groups (Table 3). When we analysed EF as a continuous variable using a logistic regression model, we found no correlation between EF and in-hospital outcomes.

## Long-term outcomes

Table 4 represents the unadjusted estimates of adverse event rates in the long-term follow-up after discharge from hospital, using the Kaplan Meier method to account for right censoring. A statistically significant difference was observed in the Kaplan-Meier estimates from groups 1 to 3 for only cardiac death (2, 0.4 and 0.3%, *p* = 0.004). No such trend was present for cumulative MACE (5.8, 2.2 and 3.3%, *p* = 0.1) or for TVR, TLR and CABG. Table 4 shows the Kaplan Meier estimates for adverse clinical outcomes in follow-up.

**Table 4.** The Unadjusted Long-Term Outcomes Of Intervention In Patients With EF ≤ 40%, EF 5 41−49% And EF ≥ 50%, Using Kaplan-Meier Estimates

	*Group 1 (EF ≤ 40%; n = 293)*	*Group 2 (EF = 41−49%; n = 268)*	*Group 3 (EF ≥ 50%; n = 1469)*	
Variable	n (%)	n (%)	n (%)	p-value
Cardiac death	5 (2)	1 (0.4)	4 (0.3)	**0.004**
Non-fatal MI	1 (0.4)	0	11 (0.8)	0.31
TVR	9 (3.5)	4 (1.7)	31 (2.3)	0.58
TLR	5 (1.9)	2 (0.9)	13 (1.0)	0.38
CABG	6 (2.3)	4 (1.7)	22 (1.7)	0.95
Cumulative MACE	15 (5.8)	5 (2.2)	43 (3.3)	0.1

MI: myocardial infarction, TVR: target vessel revascularisation, TLR: target lesion revascularisation, CABG: coronary artery bypass grafting, MACE: major adverse cardiac events.

Finally, multivariate Cox regression models were used to determine the independent predictors of long-term outcomes including cardiac death and MACE. After adjusting for potential confounding factors with p-values ≤ 0.15, patients in the lowest versus highest EF group were at increased risk of cardiac death and cumulative MACE (Table 5). Conversely, there was no significant difference in trend in the rate of these outcomes in group 2 versus 3.

**Table 5.** The Adjusted Hazards Ratios And 95% Confidence Intervals For Events In Long-Term Follow-Up

	*Group 1 vs group 3*			*Group 2 vs group 3*		
*Variable*	*Adjusted hazards ratio*	*95% CI*	*p-value*	*Adjusted hazards ratio*	*95% CI*	*p-value*
Cardiac death	5.49	1.29−23.3	0.02	0.84	0.13−11.52	0.66
Cumulative MACE	2.07	1.03−4.16	0.04	0.78	0.30–2.00	0.60

MACE: major adverse cardiac events.

Use of drug-eluting stents tended to be associated with a lower risk of MACE (HR = 0.4, 95% CI = 0.15−1.07). PCI in the left anterior descending artery territory increased the probability for MACE (HR = 2.38, 95% CI = 1.24−4.56) and TVR (HR = 3.03, 95% CI = 1.31−7.03). By analysing EF as a continuous variable, a lower EF was shown to be associated with a higher risk of MACE and cardiac death at long-term followup (HR = 0.96, 95% CI = 0.94−0.99; HR = 0.92, 95% CI = 0.87−0.98, respectively).

In the subgroup study of patients treated with stents, patients with the lowest versus highest EF category had significantly higher rates of MACE and cardiac death (HR = 2.52, 95% CI = 1.23−5.18, HR = 5.75, 95% CI = 1.35−24.56, respectively). Survival rates were 94.1, 97.4 and 96.1% in the lowest, intermediate and highest EF groups, respectively (*p* = 0.02).

## Discussion

The finding that emerged from this study is that patients with significant LV dysfunction (EF ≤ 40%) had increased rates of cardiac death and cumulative MACE in long-term follow-up compared to those with EF ≥ 50%.

Previous studies have shown that outcomes of PCI in patients with LV dysfunction were poorer compared to those who had normal EF.^7^-^13^ However, most of these data have included only the outcomes of PTCA in an era predating the current catheter-based practice. The authors of one study showed that in patients with depressed LV function (EF ≤ 35%), PTCA may be performed with low in-hospital complications and LVEF was not an independent predictor of long-term outcomes.^22^ On the other hand, in a more recent study,^23^ the in-hospital mortality and the composite endpoints of death/MI were more frequent in the lowest EF group: the in-hospital mortality rate was 3% compared to the cohort of Lindsay (2.6%)^24^ and Marisco^22^ (1.6%).

In our study, however, there was no statistically significant trend across the three groups for in-hospital cardiac death and MI, nor were there any trends across the groups for acute success rates. It seems that with the advent of stents, the in-hospital outcomes have improved dramatically. For example, while in the early 1990s, the in-hospital cardiac mortality rate in patients with severe LV dysfunction has been reported to be as high as 7%;^7^,^25^ these rates in the recent decade have reduced to zero, as seen in a cohort that used absolute stenting techniques.^26^ In a similar study, which performed stenting in 80 patients with EF < 30%, the acute in-hospital MI was 1%^16^ compared to 0.4% in our study.

The other aim of our study was to compare the rate of MACE in these three groups. In multivariate analysis, the rate of both cardiac death and MACE differed between groups 1 and 3. Similar to our results, the authors of another study have shown that the single and composite endpoints of death/MI and CABG were significantly higher in the lowest versus highest EF groups. In this study, the death rate in the lowest EF group was 11% and the actuarial survival rates were 79.1 to 88.2% in the lowest to highest EF groups.^23^ On the other hand, Holmes has shown that the four-year composite endpoint of death/MI/CABG was not significantly different in patients treated with PTCA who had EF < 45% versus EF ≥ 45%, and the four-year rate of death in patients with EF < 45% was 13%.^11^

Prior to these studies, the rate of one-year death was reported between 13 and 21%.^7^-^13^ We observed a cardiac death rate of 2% in the lowest EF group and the MACE-free survival rates were 94.3 to 96.3%. One of the reasons for the higher survival and lower cardiac death rates in our study was that we only considered cardiac deaths and not deaths from any cause. The other reason was specifically due to the higher frequency of stent use in our study.

The outcomes of stenting in patients with LV dysfunction have not been widely reported. To assess these effects in relation to the use of stents, we further conducted a study on a subgroup of patients treated with only stents. This time, the analysis showed that even MACE-free survival rates were significantly lower in patients treated with stents (94.1 vs 97.4% and 96.1%, *p* = 0.02). The EF category 1 versus 2 was again the independent predictor for MACE and cardiac death. The rates of cardiac death and MACE in this population were 2.3 and 6%, respectively.

The authors of two studies reported cardiac death rates of 6 and 12%^16^,^26^ in similar populations with depressed LV function treated with coronary stenting. In the first study, 80% of patients remained alive and free from angina, MI, CABG or repeat PCI. Follow-up events were observed more frequently in patients with incomplete revascularisation and lower EF. However, both of these studies were descriptive reports and did not provide a comparison with other EF groups.

Ultimately, while the study has shown fewer target-vessel revascularisation and cardiac event rates with CABG compared to PCI in long-term follow-up,^27^ it is not clear whether stenting can reasonably be considered an equivalent therapeutic strategy to surgery in patients with low EF or in diabetics.^14^,^15^ A prospective, controlled trial with defined criteria for treatment assignments is warranted to confirm these results.

## Conclusions

Patients with lower EF have similar in-hospital outcomes and higher long-term major adverse cardiac events and cardiac death rates after percutaneous coronary intervention. Even the use of newer techniques such as stenting may not compensate for this. In fact, the use of new stenting strategies has reduced the overall rate of these outcomes in the total population.

One of the limitations of this study was that 10.5% of patients were missed in follow-up. Although our study provided long-term evaluation of outcomes in patients, controlled analysis of these patients for a longer duration is required.
